# Epidemics, Endemics, and Contagions

**Published:** 1899-11

**Authors:** W. L. Morgan

**Affiliations:** Baltimore, Md.


### EPIDEMICS, ENDEMICS, AND CONTAGIONS.

W. L. Morgan, M. D., Baltimore, Md.

In a former paper entitled, "That Morbid Vital Force From Without Inimical to Life: What It Is?" I showed that the inter-atomic elastic ether is the force which holds the atoms of matter together, forms, gives shape and distinguishing characteristics to each and every species and kind of organic and inorganic matter, by which they are distinguished from each other.

I showed that this force is of infinite variety of kinds, equal to the number of the various kinds and species of matter, each one controlling its own species, that the life-force within the seed directs new growth after the fashion of the parent stalk; that when men or animals imbibe too much of the vital ether that is designed for some poisonous plant, it will displace or obstruct the natural vitality and cause sickness or death; that when we take the record of the symptoms of a sick person, they lead us to find some plant or other substance that is known to have produced similar symptoms in a healthy person, and that, if it is a plant, we may conclude that it has collected from the surroundings the similar vital ether which directed its growth, and know that it is the similiar and curative remedy for the case; that the decomposing processes of nature serve to break up the molecular formation of matter and set free this inter-elastic vitality; that the restless agitation of air and water serves to attenuate and expand it to a degree suitable for reformation into new generations of similar species; and that it may miscarry and be taken up into other susceptible organisms where its influence is more blighting than nourishing.

With a knowledge of the above causes of the efficacy of high potencies in provings and in the treatment of the sick, as a basis for logical reasoning as to, "What is cause, and what causes produce certain effects?" we may by such reasoning and deductions, arrive at a proper understanding of the origin, movement, and spread of epidemics and endemics, their relation to contagions, and their phenomena and characteristics.

All malarial and miasmatic diseases are endemic in character, as they are confined to certain localities, generally where there are low grounds and swampy places, where there is much vegetation in the various stages of decomposition, or where there are large tracts of level country along the margin of rivers and lakes, with fertile soil and a rich growth of vegetation.

During the rainy season, the streams overflow, the low lands are flooded, remaining inundated for many days. Finally the sediment settles and forms a mud-covering over the vegetation, often becoming many inches thick before the water runs off. This becomes dry and attains various degrees of heat from the sun and there result fermentation, decomposition, and the generation of fungoid growths of mold in the vegetable mass below, the baking process having gone on for some time. The mud covering cracks opens in seams, through which the noxious gasses, miasms, and disease-producing elements of various kinds rise, according to time, quality, and kind of vegetation, depth of covering, degree of temperature, stage of decomposition, etc. These floods generally occur in the spring or early summer, leaving the hottest part of the season for the heating and fermenting process. This is liable to fill the air of large districts with these poisonous vapors for several months at a time. The miasms may be carried by the winds for many miles in all directions, and although they may become highly diluted or mixed with air, we may suppose even equal to the two-hundredth or one-thousandth potency, yet they are still sufficiently strong to leave disastrous diseases in their track. In fact, strong, regular winds, blowing over an extensive district of fermenting hot-beds, can throw these miasms hundreds of miles over the land and afflict, for a season, the inhabitants of an otherwise healthy locality. These cases occur sporadically, the victims having had no intercourse with parties from any other place, and yet they are found to have all the symptoms of the cases at the home of the miasm. This gives an excellent example of the disease-producing power of miasms, even though so far from home and so highly attenuated with such large quantities of air. However, the susceptibility to these more severe forms of fever may be augmented by the milder miasms of the locality. From these observations, we conclude that epidemic, endemic, and sporadic malarial diseases are all from the same source, only in a different degree of intensity or potency, made by nature's machine (atmospheric attenuation), similar to the method employed by the homœopathic pharmaceutist in preparing his high potencies. In the higher and cooler latitudes and altitudes, these miasmatic conditions cause agues and intermittent, remittent, bilious, malarial, and typhoid fevers, while in the southern latitudes or tropics, we find cholera, dengue, and yellow fever resulting.

The epidemics of the hot climates being explained as above, we come next to the higher mountainous districts and colder regions, where we find an entirely different class of diseases prevailing. Here such diseases as pneumonia, typhoid fever, diphtheria, measles, and scarlet fever become epidemic. Only the last two, however, are contagious, and, although quite different in characteristics, they originate from a similar, though different miasm. In the higher mountain regions, we find the country covered with heavy forests, in which there are many dead and rotting trees. This state of affairs is also found in the rolling and hilly districts, where there are deep ravines. In such places, the leaves fall in the autumn and mingle with the fallen trunks and branches of the dead trees. They remain soaked with the rain and the snow until the heat of the next summer, when the new leaves form a shade and prevent the sun from drying the undergrowth. This vast field of dead vegetation is in a continued state of moisture, but not submerged with water, nor covered with mud, neither is it subjected to the sweating or baking process like that of the low or level lands. In consequence, the rotting or decomposing processes are much slower. In these shaded and damp places, large amounts of dead vegetation are always found, especially wood, bark, and leaves. There is always found a great variety of mosses and fungoid growths, which in turn die and contribute to other new growths of vegetation; the fungoid growths are those large and small, hard and soft, pasty and jelly-like substances, which exude from the dead and rotting wood and bark of trees, adhering to the outer surface as a new covering. Here allow me to call the attention of any one who has had the opportunity of seeing both, to the near resemblance this fungoid growth has to the exudation from the devitalized or dead tissues of the diphtheritic membrane in the throat of a child suffering with that disease. Now I will ask such a one to think quietly for a few minutes, before condemning this comparison. Collect from your memory these facts: In such localities and where such conditions exist, you always find the greatest virulence of that disease; wherever diphtheria and typhoid fever exist, you always find decomposing or rotting vegetation somewhere near by or to windward of the place where the disease was contracted; that this same kind of fungus, which is found on the dead bark and wood and on the diseased limbs of living trees is composed largely of a growth of the same appearance as that which is found on the diseased tissues of a living child's throat. So you see, that the idea that the two growths come from the same conditions and causes is not so far-fetched. It is well to keep in mind, that all molds seen upon damp things in the house are only another form of fungus growth.

From these observations, we should understand the reason why malarial and intermittent diseases are found in low, swampy places, where the rotting processes go on under water and mud, and why the inflammatory and exanthematous diseases are more common in the higher or mountainous regions, where the rotting substances are not mixed with standing water, but with the clearest of rain water, and are free from hot air.

That these emanations from disease-producing forces should cause sickness in the near vicinity, is easy to understand, but remember, they can also be driven by the regular wind currents, continuing for a long time in one direction, to very long distances, and being highly potentiated and mingled with the air, they are able to disturb the vital harmony of many people over large districts of country. Then for a few weeks and sometimes for a few months an epidemic prevails. But some day it will suddenly disappear as mysteriously as it came, simply because the direction of the wind has changed, although this is not noticed by any but close observers.

In this way, the two kinds of miasms may mingle together in the winds and afflict the people of a territory far from the home of either, causing depressing effects and sicknesses of a mixed character, such as grippe, which may have the characteristics of many diseases in the patient at the same time. That grippe should be considered to be the result or product of a combination of miasms, ought not to be considered strange by physicians who practice by symptoms. In analyzing a common case, we find it commences suddenly with the chill, fever and sweat of the swamp miasm, the aching, strained feeling of the dengue, and the bilious symptoms of the tropical fevers, the slow remittent fever and tired-out feeling of the chest, lungs, and abdomen of the mountain typhoid, the sore throat and cough of diphtheria, and the pneumonia of the higher countries. It is not confined to any locality, latitude, altitude, land, sea or season, and travels too fast for any motive power except electricity. Hence we can form no other opinion with logical reason, than that it is epidemic from combined miasms from long distances and far apart, highly attenuated with atmospheric air and highly potentiated by the winds and storms. Then just like high potencies, it acts quickly and with deep and lasting effect, and when taken in too frequent doses by a susceptible subject, makes itself severely felt.

The grippe has not yet been successfully proven by clinical experience to be contagious. However, in this enlightened age some microscopist will soon discover a suitable microbe and soon after some M. D., Ph. D., etc., will take a scab from a mangy dog of the swamp regions of the south and inoculate a jack-rabbit of Colorado and get a serum. He will then in turn inoculate a few people with it and publish abroad that, "It is a sure preventer of grippe, because hundreds of people were inoculated and have not had the grippe since." At once the combined health boards will apply to the legislatures and have grippe made contagious by act of assembly. Then they will have money appropriated, with which to enforce the law. This will make it highly contagious; it will be no longer epidemic.

Another very powerful factor in making up the list of causes of epidemic diseases is found in the influence of clouds and the sun's rays upon the vitality, when the clouds approach near the surface of the earth. The light on reaching the clouds is subject to the natural laws of refraction. The bright, warmer, and vitalizing rays are refracted and turned away, while the dark or X-rays pass directly through to the earth and like the rays from the Crookes tubes, have a blighting or killing influence on both animal and vegetable life. By reason of cloudy weather of long continuance, we often hear the remarks, that vegetation will not grow, but looks pale and sickly, and that animals in the fields look dull and drooping. Little does the chronic rheumatic think, when he dreads the approach of cloudy weather on account of the severe pain it causes, that it is the X-rays on a large scale, with moderate effect, doing the kind of work that they will do in a condensed and more violent way from the Crookes tube. While the cloud rays make a man melancholy and remind him of all the misdeeds and misfortunes of his past life, the rays concentrated and sent out from the tube penetrate flesh and blood, and, continued long, will forcibly remind him of the torments in the future life for the disobedient, for which statement, I refer you to the provings made by Dr. Finke and published in HOMŒOPATHIC PHYSICIAN, and confirmed by myself and others. Add to all these effects of both cloudy and stormy weather, the low barometer, and the thin air heavily charged with miasms and deficient in vital nourishment, and you will well understand how under such conditions, the sick persons should succumb and the well ones become sick. You will well understand how the vitality becomes so deranged as to be unably to rally, when better conditions prevail, until after a long and wearisome sickness, or indeed so deep may be its influence, that its baneful effects may be fatal or disturb the health during the remainder of life.

Fogs arising from the moist lands and forests, no doubt contribute to the toxic effect of the miasms, but running water, which is supposed to be a dangerous carrier of disease elements, makes a two-sided subject, with many evidences for the negative. All streams run over either earthy, sandy or rocky bottoms. In the smaller branches, all the water comes in contact with the earthy substance and it is well known that earth and sand are the greatest absorbents of water contaminations and are the best purifiers of water known. At the same time water is known to absorb malarial miasms. A paludal district to windward of a large sheet of water is troubled with intermittent and other malarial diseases, while those districts on the leeward side are seldom or never visited by such fevers. This is evidence that the water absorbs the disease miasms from the wind as they pass over it. This is from old history of agues with recent observations. Just view the situation in the Allegheny mountains. On the western slope, the Monongahela river rises in West Virginia, it flows northward until it meets the Allegheny at Pittsburg, where they form the Ohio, which flows west and south. Typhoid fever and diphtheria are the prevailing diseases high up on the tributaries of both rivers. In these districts the per cent. of cases is much greater than in Pittsburg, where all the wash from all the cases of all the country flows into the city and is pumped into the reservoirs for washing, drinking, and all other purposes. Now this same water, plus all the sewerage of Pittsburg and all the filth gathered in its course down the Ohio, is pumped into the reservoirs at Cincinnati to be used for the same purpose. Yet in spite of all this additional dirt and filth, there is a less percentage of typhoid and diphtheria there than at Pittsburg, and I think these same observations may be made of the Potomac River in regard to Cumberland and Washington. With these and many other references, which might be mentioned, we must conclude that the preponderance of evidence is that wherever water contamination is supposed to be the cause of the spread of disease, a careful investigation will prove that it comes instead from some form of rotting vegetable matter near by; the second, third, or any number of cases coming after the first, either in the same house or near by, are from the same source as the first, and, in the cases of diphtheria and typhoid fever, are not transmitted from one to another, the disease is endemic, a miasm in the house or neighborhood, and can only be remedied by a general cleaning and renovating of the premises, and removing from the surroundings all damp or rotting vegetation of every kind.

When the water of a well is supposed to be the cause of sickness, it will be effectually remedied by taking away all moss, old rotting wood, and other kinds of rotting vegetable or animal matter, cleaning out the contaminated water and mud, washing every part clean, finishing the top with cemented brick or stone, and supplying an iron pump or new bucket. The new supply of water will be pure and good, for there will be no rotting or molding substances to produce disease-generating miasms or food for the supposed-to-be-dangerous microbes to live on, and they will not come again till there is another supply of rotten stuff to make food for them. Rotting animal matter combined with decaying vegetation is a prolific source of yellow fever miasm, as was proved by Gen. B. Butler's sanitary orders when in command at New Orleans.

### DIPHTHERIA IN A DRY COUNTRY.

Since writing the above, I received a letter from R. A. Blackburn, M. D., of Sutton, Nebraska, describing an epidemic of diphtheria in western Nebraska, an exceedingly dry country, stating that it was worse in winter and after a heavy snow or blizzard. And also a statement from Dr. H. Peters from northern New York, near the Hudson river, that in his experience he had found it worse in winter and after the first heavy snow. Both ask how these facts harmonize with my theories as expressed in the paper on diphtheria, published in the *Hahnemannian Advocate*.

I think these judicious inquiries are answered in the above, where I explain that the miasms rising from the swamps, fields, and forests where they are generated, are carried along with the fogs by the winds to very great distances. The fogs become frozen into snow and descend to the ground in an otherwise healthy country and contaminate the air and surface soil. With these influences combine the extreme changes of temperature and the blighting influence of the cloudy weather and dark rays, or X-rays. All those depressing conditions are at any time liable to distune the life-force and render the system susceptible to the ravages of the epidemic miasms, imported and intensified by the snow, and spread serious sickness over territories, otherwise healthy, even thousands of miles from where it was generated.

While these reasons are entitled to consideration, there may be other causes and sources that may also create and spread diseases that cannot be so easily traced.

